# Supplementary material for: Apelin Resistance Contributes to Muscle Loss during Cancer Cachexia in Mice
Source: Cancers (Basel). 2022 Apr 2;14(7):1814. doi: 10.3390/cancers14071814 (PMC8997437; doi:10.3390/cancers14071814)
Supplement: Supplementary file 1 [file cancers-14-01814-s001.zip › cancers-1608741-supplementary.pdf]

Supplementary Materials: Apelin Resistance Contributes to Muscle Loss during Cancer Cachexia in Mice

Andrea David Re Cecconi, Mara Barone, Mara Forti, Martina Lunardi, Alfredo Cagnotto, Mario Salmona, Davide Olivari, Lorena Zentilin, Andrea Resovi, Perla Persichitti, Dorina Belotti, Federica Palo, Nobuyuki Takakura, Hiroyasu Kidoya and Rosanna Piccirillo

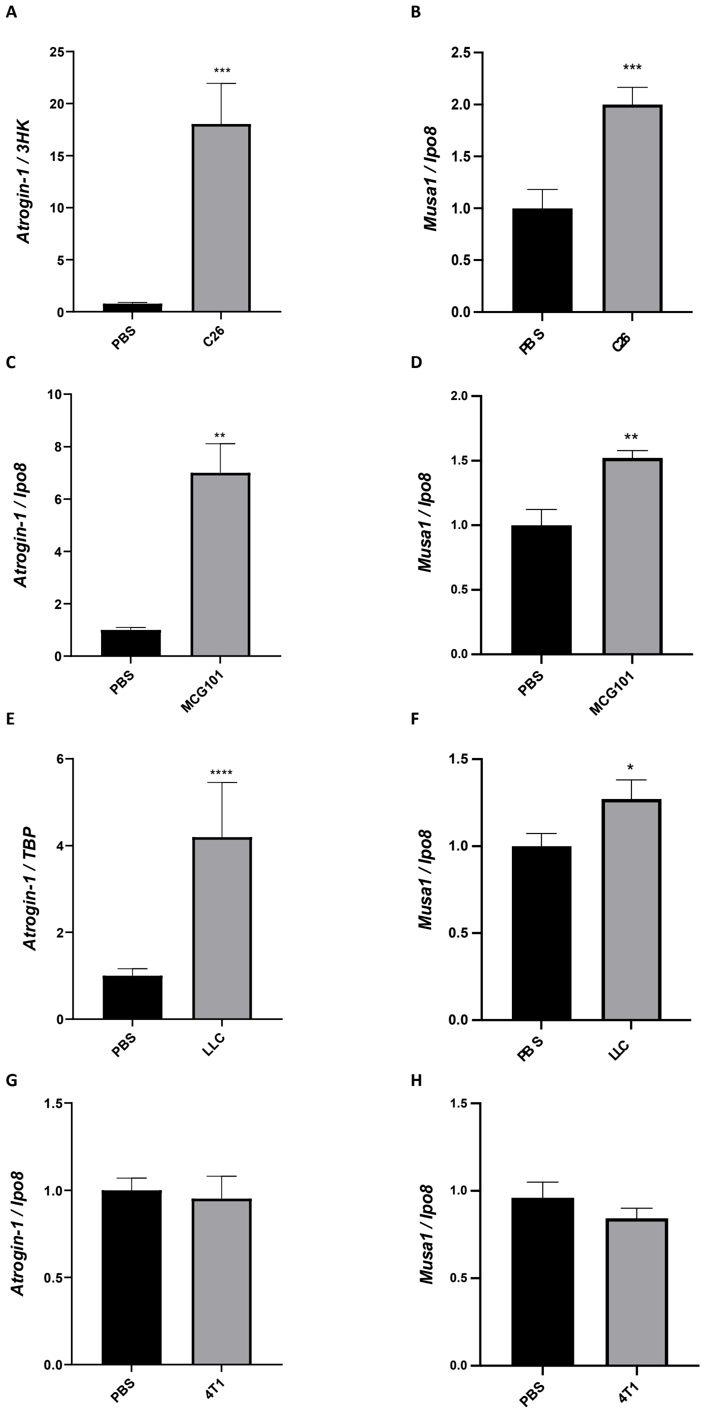

Figure S1. In the atrophied muscle of the three animal models of cancer cachexia, the expression of *Atrogin-1* and *Musa1* increase. Q-PCR datasets show that mRNA levels of the ubiquitin ligase Atrogin-1 and Musa1 are induced in the TA of mice carrying the tumor C26 (**A–B**), MCG101 (**C–D**) or LLC (**E–F**). Unpaired *t*-test , * $p \leq 0.05$, ** $p \leq 0.01$, *** $p \leq 0.001$, **** $p \leq 0.0001$; n = 7–10 (**A–F**). The mRNA levels of *Atrogin-1* and *Musa1* are not induced in the TA of mice carrying the non-cachectic tumor 4T1 (**G–H**). Unpaired *t*-test, not significant, n = 5. 3HK, three housekeeping genes (*TBP*, *Ipo8* and *Gusb*), Ipo8, Importin 8 and 18S, 18S ribosomal RNA, were used to normalize the data.

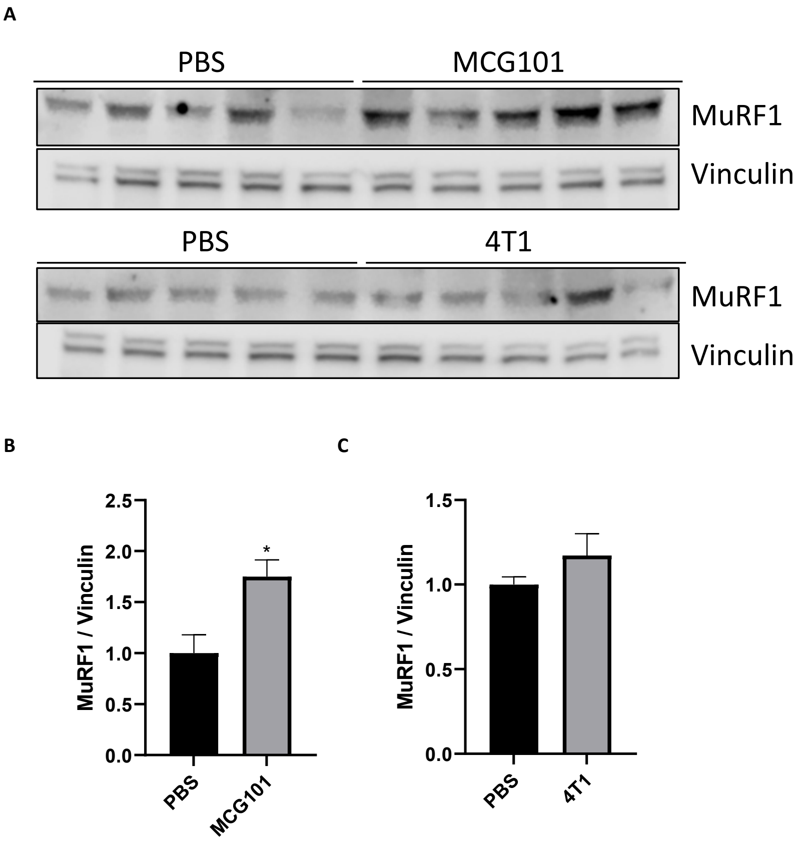

Figure S2. MuRF1 protein levels increase only in the atrophied TA of MCG101 model of cancer cachexia and not in the non-cachectic TA from 4T1 carriers. Proteins from TA muscles of MCG101- and 4T1-carrying mice were analyzed in Western Blot for MuRF1 and vinculin as loading control (**A**). Quantitations for MuRF1/vinculin in MCG101- (**B**) and 4T1-bearing mice (**C**) are shown. Unpaired *t*-test , * $p \leq 0{,}05$, n = 5.

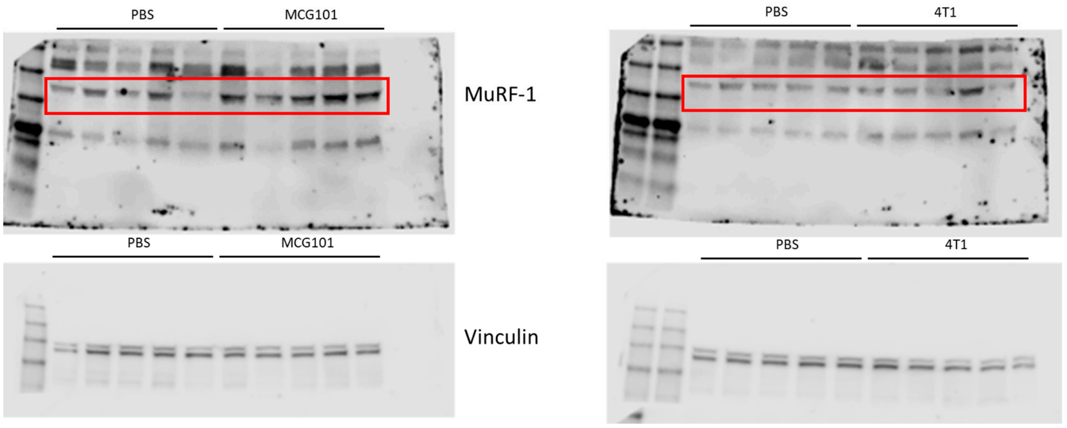

Figure S3. Uncropped western blots of Figure S2A.

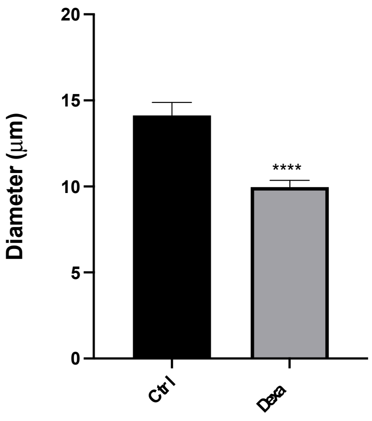

Figure S4. Twenty-four hour-treatment with dexamethasone reduces myotube diameter by about 30%. Ten µM dexamethasone treatment for 24h on 4 days-differentiated myotubes reduces their cell diameter, as compared to vehicle (ctrl, H_2O). Dexamethasone, Dexa. Unpaired *t*-test, **** $p \leq 0.0001$, $n = 6$.

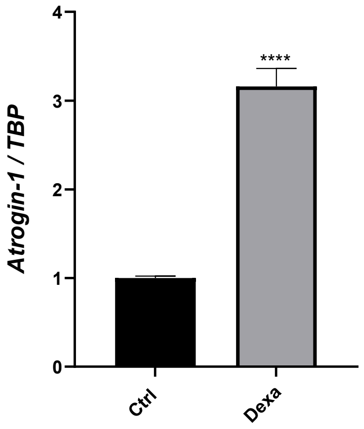

Figure S5. *Atrogin-1* is induced by dexamethasone in myotubes. Four day-differentiated myotubes were exposed for 24h to vehicle (Ctrl) or 10 µM dexamethasone (Dexa) and the Q-PCR dataset shows that *Atrogin-1* expression is induced. *TBP* was used as housekeeping gene. Unpaired *t*-test, **** $p \leq 0.0001$, $n = 6$.

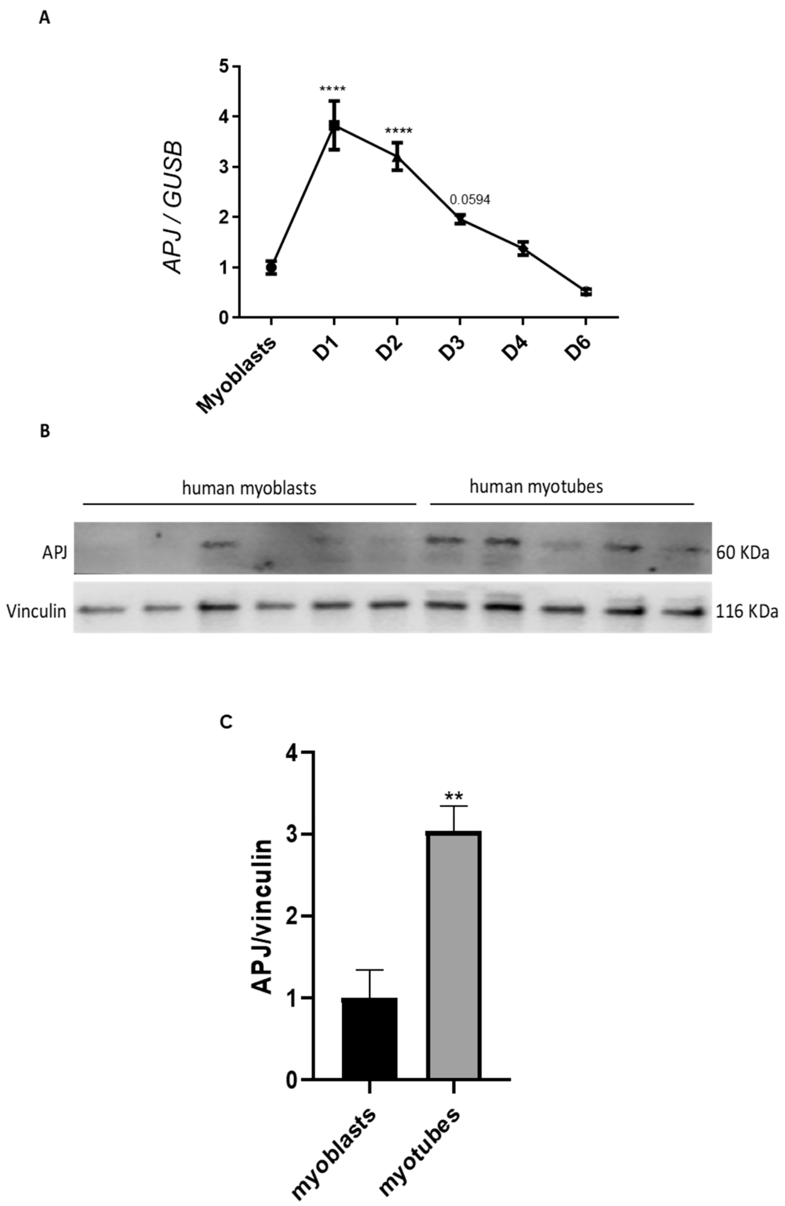

Figure S6. A*PJ* is expressed in murine and human myoblasts and during their differentiation. Q-PCR shows that mRNA levels of APJ are reduced after D1 and D2 during myoblast differentiation (D = days). Ordinary one-way ANOVA, Dunnett's multiple comparison test, **** $p \leq 0.0001$, $n = 5$ (**A**). Western blot (**B**) and relative quantitation (**C**) show that the protein content of APJ is induced in human myotubes compared to human myoblasts. Vinculin is used as loading control. Unpaired *t*-test, ** $p \leq 0.01$, $n = 5$–6.

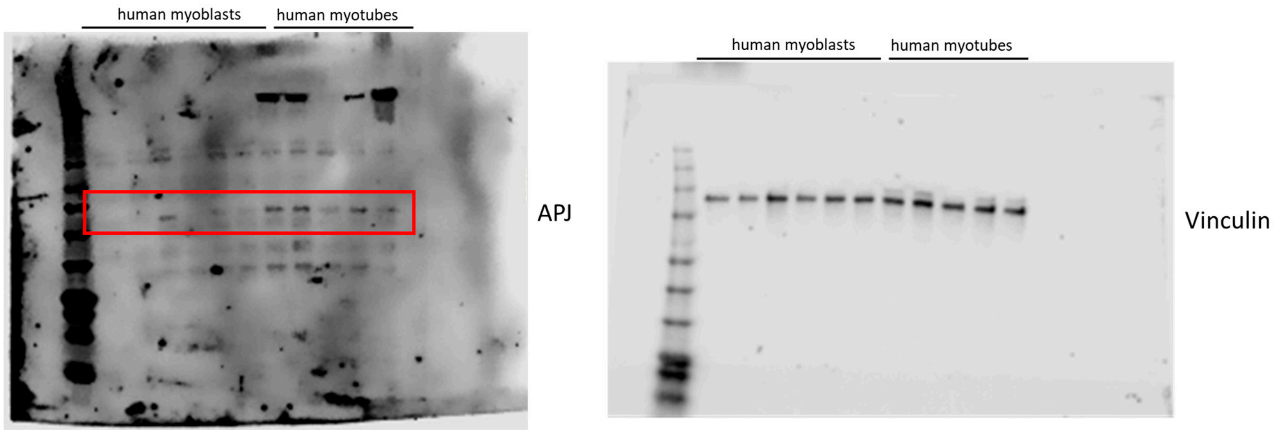

Figure S7. Uncropped western blots of Figure S6B.

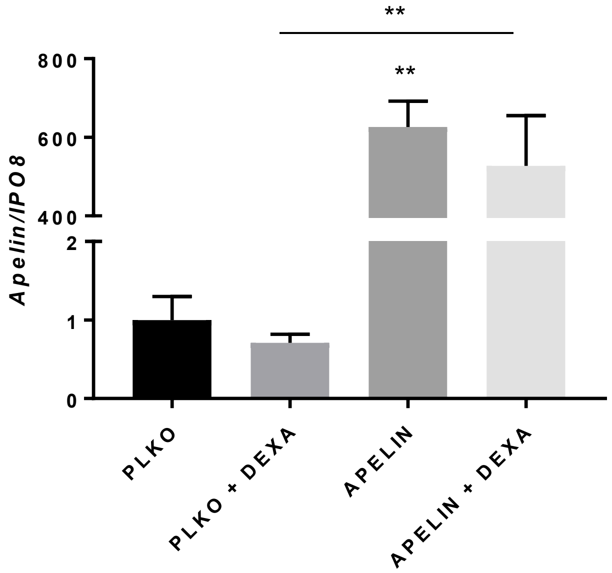

Figure S8. Twenty-four hour-transfection with preproapelin-expressing plasmids in myotubes highly increase the mRNA levels of apelin. Four days-differentiated myotubes transfected with the empty vector PLKO, as control, or preproapelin-expressing plasmid (APELIN) for 24h were treated with 10 µM dexamethasone (DEXA) for 24h. By Q-PCR, the expression level of *apelin* and *IPO8* (housekeeping gene) was measured, and the ratio plotted. Ordinary one-way ANOVA, ** $p \leq 0.01$, $n = 3$.

A

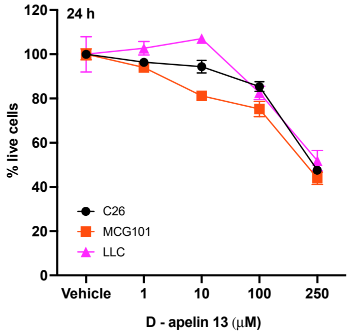

B

D - apelin 13 24 h	P-value	
Vehicle C26 vs 250 C26	****	<0,0001
Vehicle MCG101 vs 10 MCG101	*	0,0294
Vehicle MCG101 vs 100 MCG101	***	0,0005
Vehicle MCG101 vs 250 MCG101	****	<0,0001
Vehicle LLC vs 250 LLC	****	<0,0001
1 C26 vs 250 C26	****	<0,0001
1 MCG101 vs 250 MCG101	****	<0,0001
1 LLC vs 100 LLC	*	0,0364
1 LLC vs 250 LLC	****	<0,0001
10 C26 vs 250 C26	****	<0,0001
10 MCG101 vs 10 LLC	**	0,0012
10 MCG101 vs 250 MCG101	****	<0,0001
10 LLC vs 100 LLC	**	0,0029
10 LLC vs 250 LLC	****	<0,0001
100 C26 vs 250 C26	****	<0,0001
100 MCG101 vs 250 MCG101	****	<0,0001
100 LLC vs 250 LLC	****	<0,0001

C

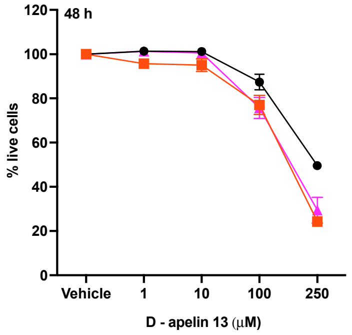

D

D - apelin 13 48 h	P-value	
Vehicle C26 vs 100 C26	*	0,0486
Vehicle C26 vs 250 C26	****	<0,0001
Vehicle MCG101 vs 100 MCG101	****	<0,0001
Vehicle MCG101 vs 250 MCG101	****	<0,0001
Vehicle LLC vs 100 LLC	****	<0,0001
Vehicle LLC vs 250 LLC	****	<0,0001
1 C26 vs 100 C26	*	0,0444
1 C26 vs 250 C26	****	<0,0001
1 MCG101 vs 100 MCG101	***	0,0008
1 MCG101 vs 250 MCG101	****	<0,0001
1 LLC vs 100 LLC	****	<0,0001
1 LLC vs 250 LLC	****	<0,0001
10 C26 vs 100 C26	*	0,0496
10 C26 vs 250 C26	****	<0,0001
10 MCG101 vs 100 MCG101	**	0,0015
10 MCG101 vs 250 MCG101	****	<0,0001
10 LLC vs 100 LLC	****	<0,0001
10 LLC vs 250 LLC	****	<0,0001
100 C26 vs 250 C26	****	<0,0001
100 MCG101 vs 250 MCG101	****	<0,0001
100 LLC vs 250 LLC	****	<0,0001
250 C26 vs 250 MCG101	****	<0,0001
250 C26 vs 250 LLC	***	0,0002

Figure S9. Only 100 and 250 μM D-Apelin 13 reduce the viability of cachectic tumor cells. Toxicity test conducted on different cachectic tumor cell lines, C26, LLC and MCG101. Cells were treated for 24 (**A**) or 48h (**C**) with vehicle (H_2O) or 1, 10, 100 or 250 μM D-apelin 13 and the percentage of living cells was evaluated through SRB assay. Two-way ANOVA, Tukey's multiple comparison test was performed and the resulting *p* values were indicated in the corresponding tables (**B** and **D**), $n = 6–9$.

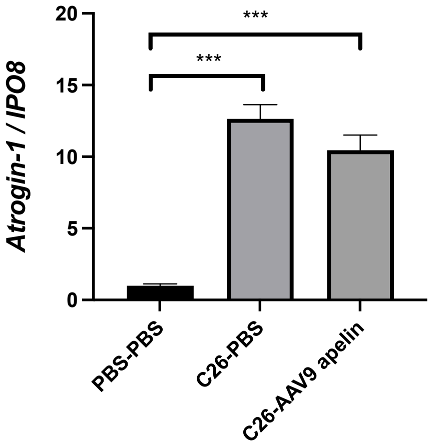

Figure S10. Atrogin-1 is not reduced by the intramuscular injection of preproapelin-expressing adenoassociated viruses. As shown by Q-PCR, the C26-induced *Atrogin-1* is not restrained by AAV9-apelin overexpression. IPO8 is used to normalize the data. Kruskal-Wallis test, *** $p \leq 0.001$, $n = 8–18$.

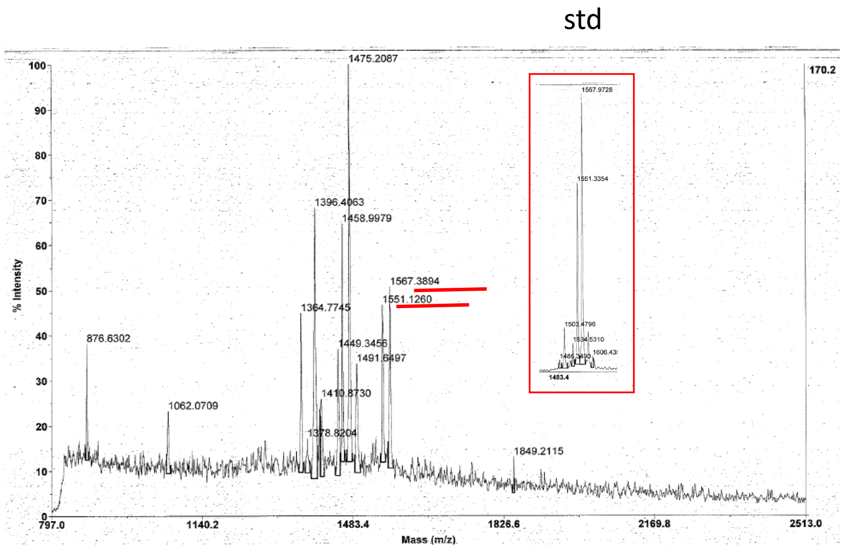

Figure S11. MALDI-TOF analysis of D-apelin 13 reveals the presence of the peptide after extraction from muscle of treated mouse. The molecular weight of 1551 corresponds to the unmodified sequence while the molecular weight of 1567 corresponds to the sequence with the methionine oxidation. The box shows the external standard, std, (D-apelin 13) briefly incubated in muscle homogenate and immediately checked using MALDI-TOF mass-spectrometer.

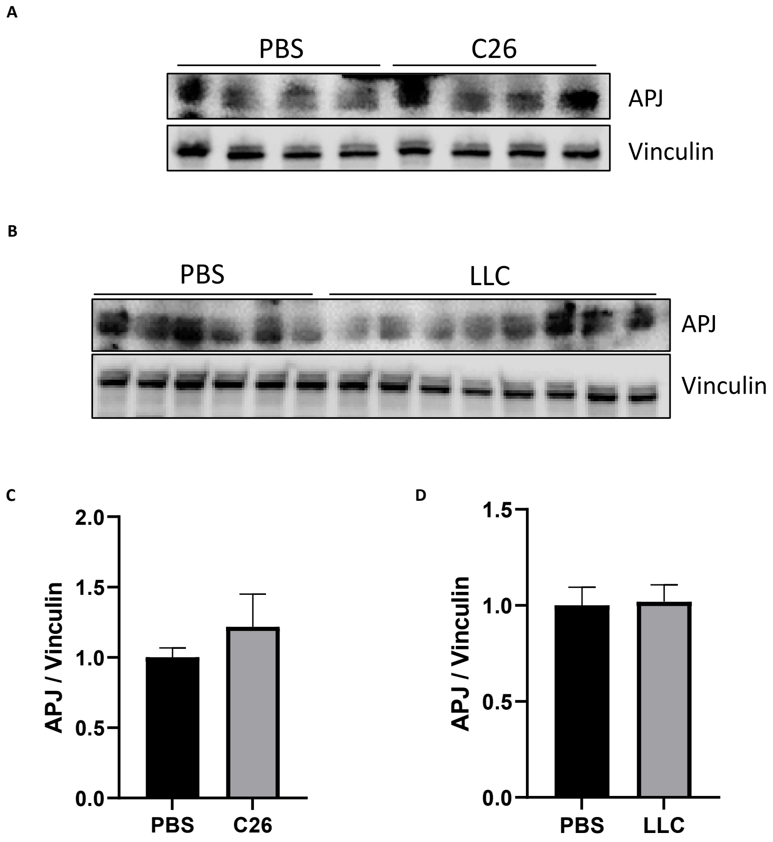

Figure S12. APJ protein content does not change in TA from two mouse models of cancer cachexia. APJ protein content does not change in TA from two mouse models of cancer cachexia. Western Blot analysis of APJ in protein extracts from TA of PBS- or C26-injected BALB/c mice (**A**) and from PBS- or LLC-injected C57BL6/J mice (**B**) and its relative quantitations (**C** and **D**) are

shown. Vinculin was used as the loading control. Mann Whitney test, not significant, $n = 4$ for (**A, C**); $n = 6$-8 for (**B, D**).

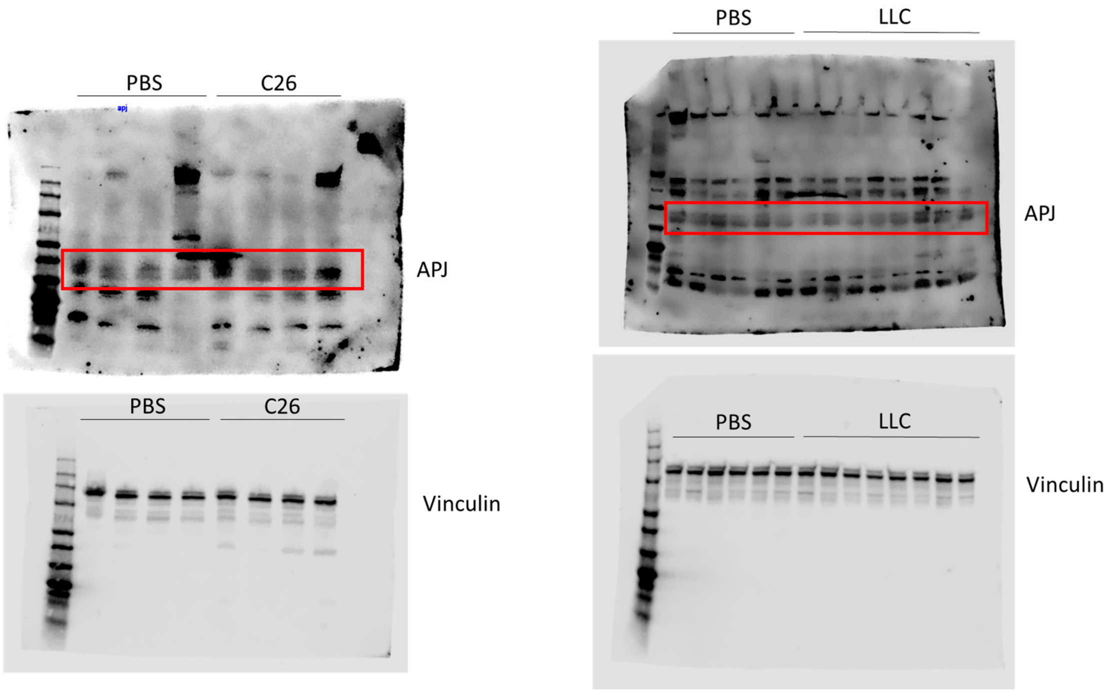

Figure S13. Uncropped western blots of Figure S12A,B.

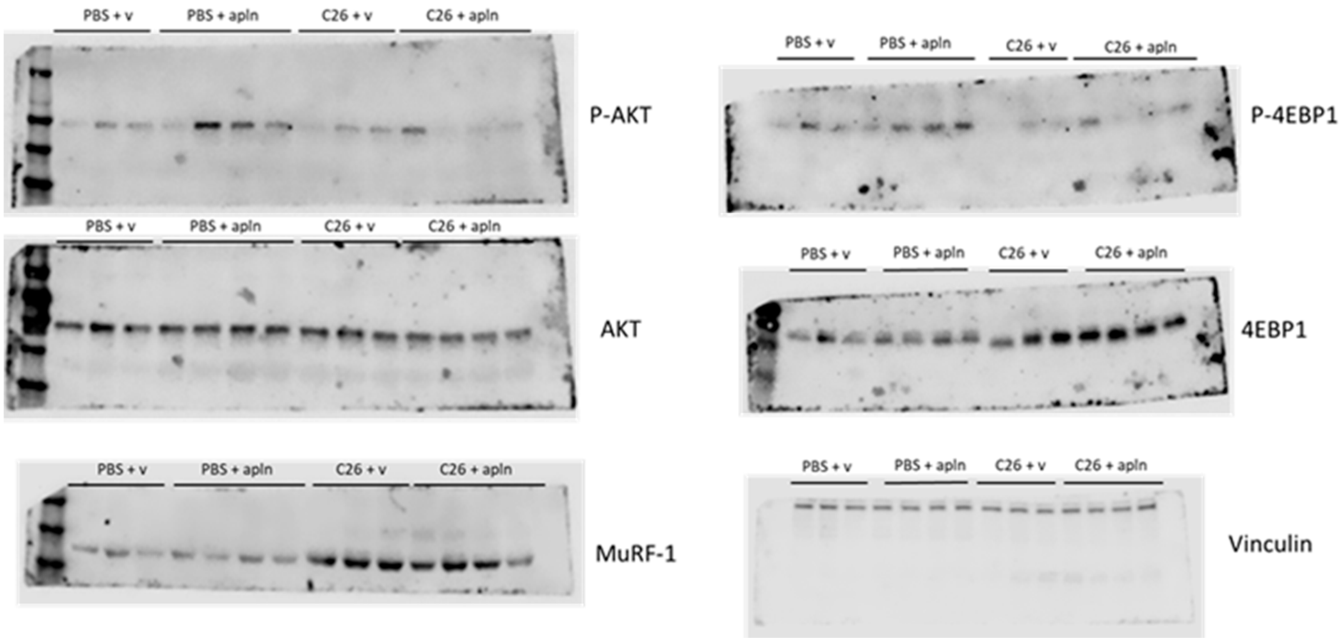

Figure S14. Uncropped western blots of Figure 6.

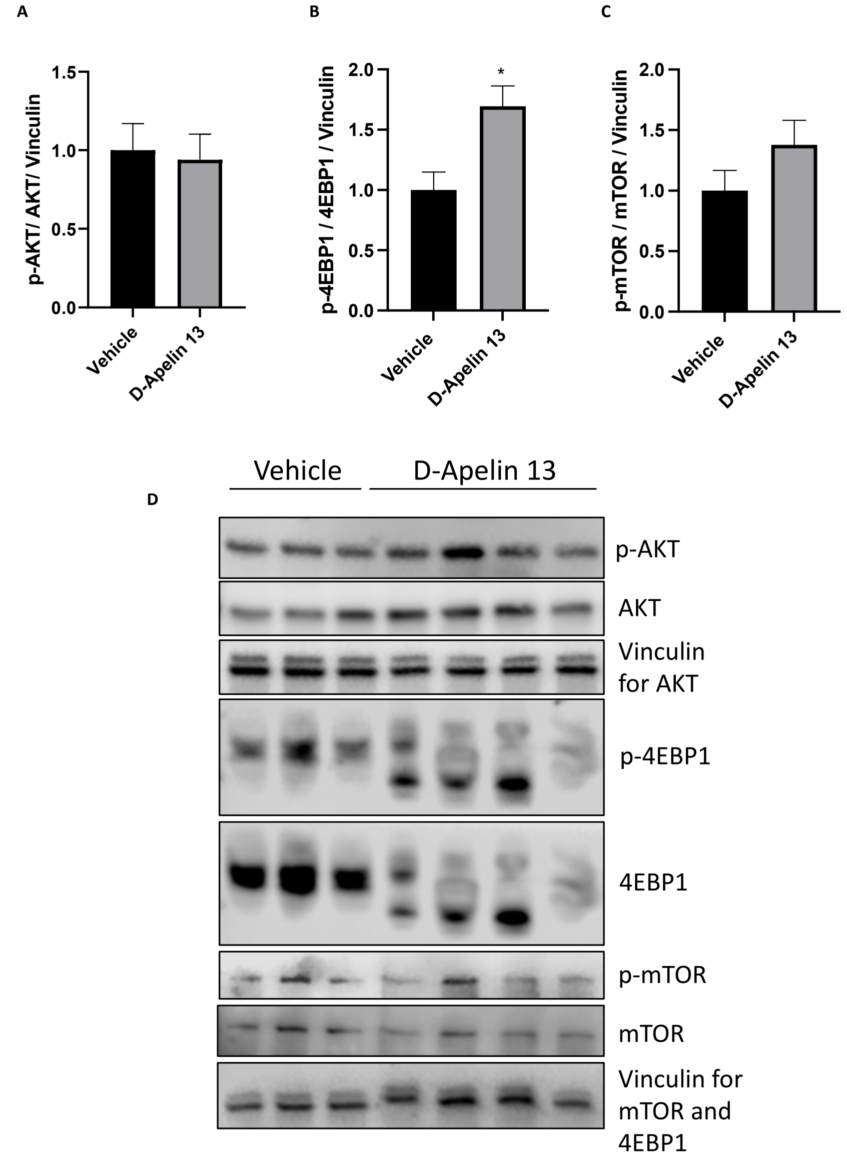

Figure S15. D-Apelin 13 treatment activates anabolic pathways in non-cachectic mice. D-Apelin 13 treatment activates anabolic pathways in not-cachectic mice. TA muscles of 9-10 week-old male BALB/c mice treated with PBS or D-Apelin 13 were analyzed using western blot for AKT (**A**), 4EBP1 (**B**) and mTOR (**C**) and their phosphorylated forms. The band quantitation is shown. Western blot analysis is exhibited in (**D**). Vinculin was used as loading control. Vehicle-treated mice served as controls. Unpaired *t*-test, * $p \leq 0.05$, n = 3-4.

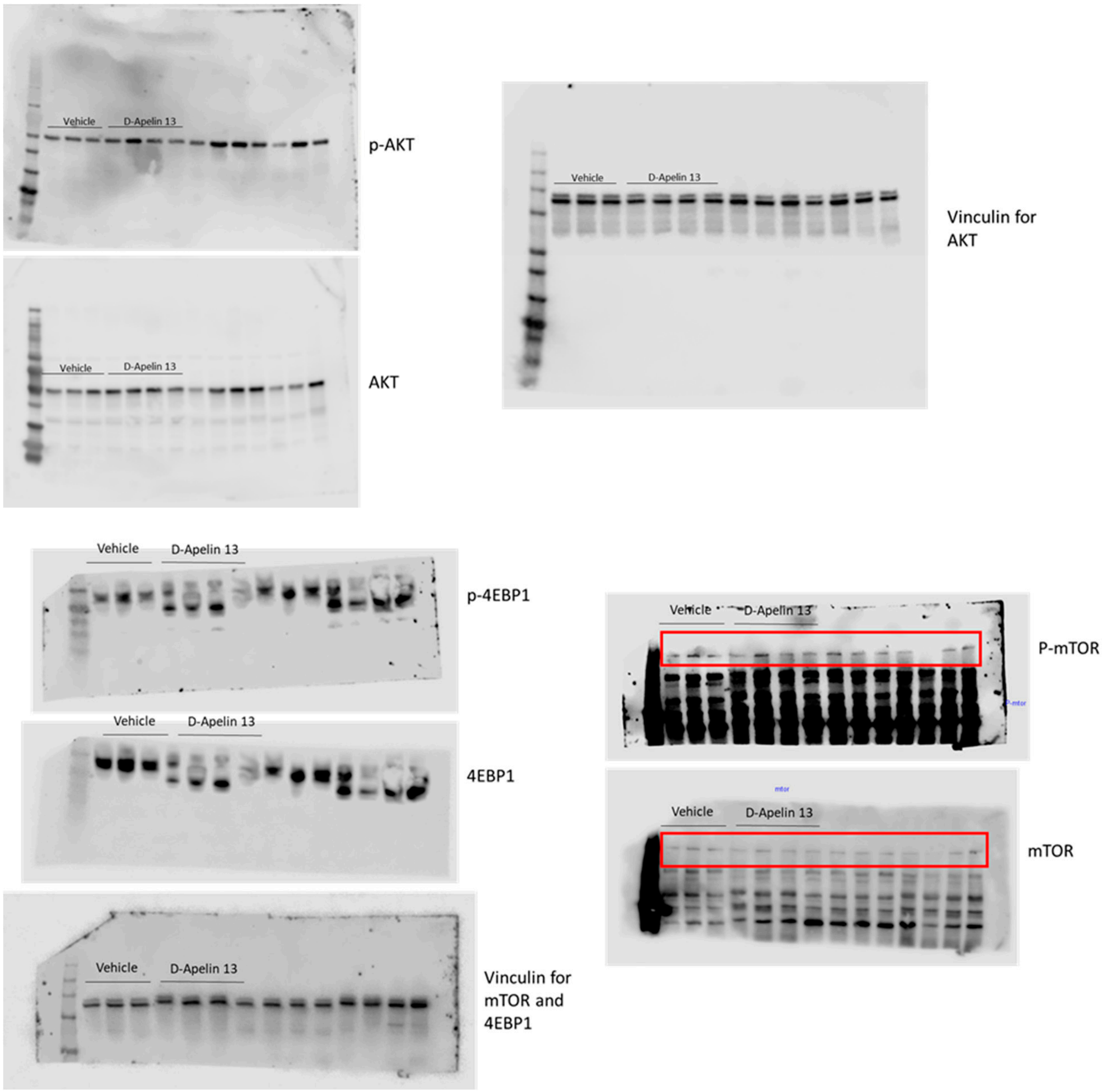

Figure S16. Uncropped western blots of Figure S15.

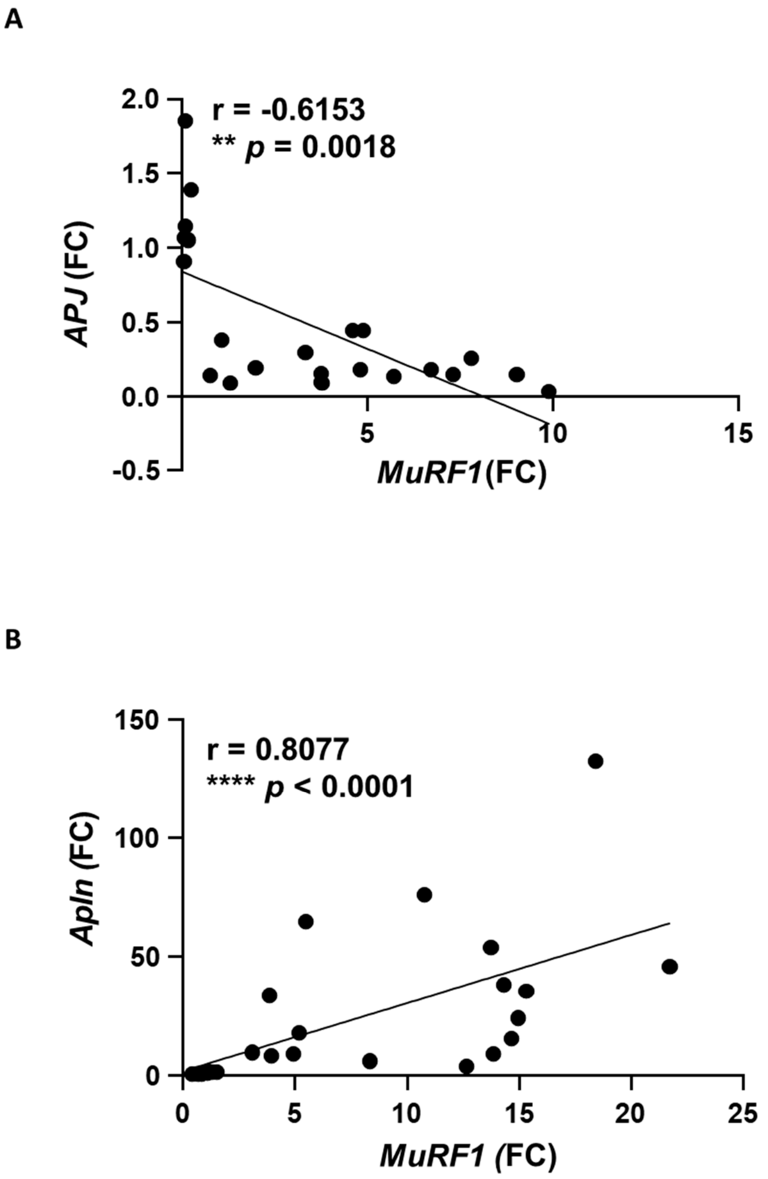

Figure S17. When over-expressed in tibialis anterior muscles, unlike *apelin*, *APJ* expression anti-correlates with *MuRF1* expression. The scatter plot shows the inverse correlation between the expression of *APJ* and *MuRF1* in TA electroporated with APJ-encoding plasmid in PBS- and C26-injected mice. Spearman's test, with correlation index (R) = - 0.6153, ** $p = 0.0018$, $n = 23$ (**A**). The scatter plot shows the correlation between the expression of *Apln* and *MuRF1* in TA of PBS-injected mice or C26-carriers both injected with preproapelin-expressing AAV9. Spearman's test, with correlation index (R) = -0.8077, **** $p \leq 0.0001$, $n = 25$ (**B**).

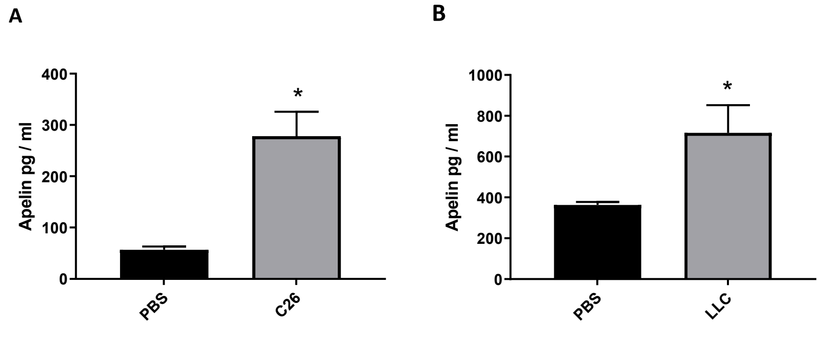

Figure S18. Plasma apelin is increased also in cachectic cancer-bearing female mice. ELISA was done on the plasma of female mice with the cachectic tumors C26 (**A**) and LLC (**B**). Apelin was high in plasma of all cancer types-bearing animals. Unpaired *t*-test, * $p \leq 0.05$. PBS, C26, $n = 3 – 5$; PBS, LLC, $n = 8 - 9$.

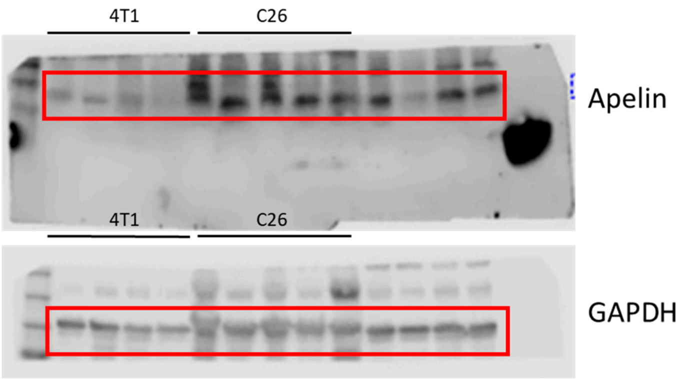

Figure S19. Uncropped western blots of Figure 7D,E.

Table S1. All the primers used in this study are listed in this table.

Gene Name	Company and Code Number	Forward Sequence	Reverse Sequence
Apln	IDT	GTCCAGTCCTCGAAGTTCTG	GTCTCTGGCTCTCCTTGACT
Apj	IDT	GCCAAGTAGCACCAAAACCTA	AATCCTCATCCCTCCACCT
Gusb	Sigma	TCGTACCAGCCACTATCCCTA	AAAACTCTGAGGTAGCACAATGC
Tbp	Sigma	ACCCCACAACTCTTCCATTCT	TTTGAAGCTGCGGTACAATTC
Ipo8	Thermofisher Mm01255158_m1	Not available	Not available
Trim63/MuRF1	Thermofisher Mm01185221_m1	Not available	Not available
Fbxo32/Atrogin-1	Thermofisher Mm00499523_m1	Not available	Not available
Fbxo30/Musal	Thermofisher Mm01191299_m1	Not available	Not available
18S	Applied Biosystem 4352655	Not available	Not available
